# Pilot satellitome analysis of the model plant, *Physcomitrellapatens*, revealed a transcribed and high-copy IGS related tandem repeat

**DOI:** 10.3897/CompCytogen.v12i4.31015

**Published:** 2018-12-13

**Authors:** Ilya Kirov, Marina Gilyok, Andrey Knyazev, Igor Fesenko

**Affiliations:** 1 Laboratory of functional genomics and proteomics of plants, Shemyakin and Ovchinnikov Institute of Bioorganic Chemistry, Moscow, Russian Federation Shemyakin and Ovchinnikov Institute of Bioorganic Chemistry Moscow Russia

**Keywords:** *
Physcomitrella patens
*, Bryophyta, satellite DNA, chromosomes, fluorescence *in situ* hybridization, long non-coding RNAs, rDNA

## Abstract

Satellite DNA (satDNA) constitutes a substantial part of eukaryotic genomes. In the last decade, it has been shown that satDNA is not an inert part of the genome and its function extends beyond the nuclear membrane. However, the number of model plant species suitable for studying the novel horizons of satDNA functionality is low. Here, we explored the satellitome of the model “basal” plant, *Physcomitrellapatens* (Hedwig, 1801) Bruch & Schimper, 1849 (moss), which has a number of advantages for deep functional and evolutionary research. Using a newly developed pyTanFinder pipeline (https://github.com/Kirovez/pyTanFinder) coupled with fluorescence *in situ* hybridization (FISH), we identified five high copy number tandem repeats (TRs) occupying a long DNA array in the moss genome. The nuclear organization study revealed that two TRs had distinct locations in the moss genome, concentrating in the heterochromatin and knob-rDNA like chromatin bodies. Further genomic, epigenetic and transcriptomic analysis showed that one TR, named PpNATR76, was located in the intergenic spacer (IGS) region and transcribed into long non-coding RNAs (lncRNAs). Several specific features of PpNATR76 lncRNAs make them very similar with the recently discovered human lncRNAs, raising a number of questions for future studies. This work provides new resources for functional studies of satellitome in plants using the model organism *P.patens*, and describes a list of tandem repeats for further analysis.

## Introduction

A substantial part of eukaryotic genomes is composed of different families of repetitive elements (REs). Some REs are ancient viruses (e.g., mobile elements), whereas others are *de novo* generated sequences without a specific structure. The latter include satellites, or tandem repeats (TRs), dispersed repeats and other repeat groups. TRs are the main components of heterochromatin, centromeres and telomeres (Henikoff et al. 2001, Plohl et al. 2008). TRs are important for genome stability and integrity and play a critical role in centromere function, meiotic chromosome segregation, gene regulation, X chromosome recognition and speciation (Dernburg et al. 1996, Ferree and Barbash 2009, Jagannathan et al. 2017, Menon et al. 2014, Talbert and Henikoff 2018). The genomic organization, chromosome distribution and sequence of TRs could differ significantly between closely related species and even between chromosomes of one organism (Almeida et al. 2012; Jagannathan et al. 2017, Jo et al. 2009, Kirov et al. 2017, Lim et al. 2004, Lower et al. 2018, May et al. 2005, Plohl et al. 2008, Robledillo et al. 2018, Ruiz-Ruano et al. 2016). Because TRs can mislead the recombination machine, they can also play a negative role and be the reason for genome rearrangements (Ma and Bennetzen 2006). Surprisingly, a recent study has demonstrated that TRs are not an inert part of a genome, some TRs, including those that have intergenic spacer (IGS), telomere and centromere origins, are expressed in a cell (Chen et al. 2008, May et al. 2005, Perea-Resa and Blower 2017, Yap et al. 2018, Zhao et al. 2018). Although the functions of the so-called satRNAs are enigmatic, there is a growing body of evidence that some of them can interact with different proteins and play nuclear architectural roles (Chujo et al. 2017, Staněk and Fox 2017, Sun et al. 2017, Yap et al. 2018).

The rapid evolution and high intra-monomer identity of TRs significantly hamper their study at the genome level. TRs are often collapsed or placed into an unassembled portion of the genome (e.g. Chr0, (Saint-Oyant et al. 2018)), which significantly reduces the amount of information available to study the organization of TRs. Long-read sequencing, optical mapping and other modern techniques can help to overcome these obstacles (Jain et al. 2018, Khost et al. 2017, Lower et al. 2018, Weissensteiner et al. 2017). High-throughput methods, including methods used to identify TRs from raw NGS data, have allowed researchers to gain a deeper insight into TR evolution and abundance (Lower et al. 2018, Novák et al. 2017). In addition, information about the TRs physical location is useful for understanding the TR evolution and function as well as for the improvement of the genome assembly (Saint-Oyant et al. 2018). Molecular cytogenetic techniques such as fluorescence *in situ* hybridization (FISH) or PRINS have been applied to study the genomic organization of TRs at a chromosome level (Cuadrado and Jouve 2010, Gosden et al. 1991, Jiang and Gill 2006, Kirov et al. 2017, Pavia et al. 2014, Rosato et al. 2016, Sone et al. 1999, Xiao et al. 2017). The unique nature of TRs allows their rapid localization on the chromosomes through non-denaturating FISH (ND-FISH, (Cuadrado and Jouve 2010, Jiang and Gill 2006, Kirov et al. 2017, Pavia et al. 2014, Xiao et al. 2017)). Although it is an important tool for studying the genome organization of TRs, the application of molecular cytogenetic methods is challenging and further improvement of chromosome preparation and FISH protocols are needed for some species (Kirov et al. 2016, Rosato et al. 2016).

The latest discoveries, including the specific transcription of some TRs as satRNAs and lncRNAs, which play important roles in regulatory processes, have moved satellite DNA biology from structural genomics to functional genomics. Satellite DNA annotation has been performed for a long list of plant species, but there are only a few model plants that are suitable for deep functional studies of TRs. In addition, no model basal plants are present on this list, although they could facilitate the study of the TR evolution mechanisms on a long timescale. Here, we performed a pilot satellitome analysis of the model basal plant, the moss *Physcomitrellapatens* (Hedwig, 1801) Bruch & Schimper, 1849. It is a widely used model plant for molecular and developmental biology, evolution and biochemistry studies (van Gessel et al. 2017). The “basal” position of mosses in the land plant phylogeny makes this plant unique, bridging the gap between green algae and flowering plants (van Gessel et al. 2017). The chromosome level assembly of moss has been recently performed and different transcriptomic, epigenetic and proteomic datasets as well as tools are available (Amagai et al. 2018, Fesenko et al. 2017, Fesenko et al. 2016, Fesenko et al. 2015, Lang et al. 2005, Lang et al. 2018, Ortiz-Ramírez et al. 2016, Quatrano et al. 2007, Rensing et al. 2008, van Gessel et al. 2017). Using the newly developed pyTnaFinder pipeline (https://github.com/Kirovez/pyTanFinder), we identified five TRs that show prominent FISH signals on the nucleus and chromosomes (for two TRs). Nuclear organization revealed two TRs with distinct locations, in the heterochromatin and perinucleolar bodies. One TR, called PpNATR76, was located in the IGS of 45S rRNA genes. Using transcriptomic and genomic data, we found that PpNATR76 is transcribed into lncRNAs with unknown functions. Comparison of the distinct features of PpNATR76 organization and transcription and similarities with the recently discovered IGS-related lncRNAs in humans suggest that the transcription of a functionally important satellite containing lncRNAs from the IGS region is a conserved principle between plants and humans.

## Material and methods

### pyTanFinder development

pyTanFinder was written in python v3.6 using biopython (Cock et al. 2009) and networkx (Hagberg et al. 2008) libraries. Tandem Repeat Finder tool (Benson 1999) was run in the initial step of the pipeline followed by BLASTN (Altschul et al. 1997) similarity search between different monomers. Using similarity search data, the graphs were constructed by the networkx library (Hagberg et al. 2008) and a sequence with the maximum number of edges (hits) was selected for each graph. The most representative monomer sequence is then described according to its different features including accumulating abundance (the sum of the copy number of each monomer from graphs multiplied by the monomer length), monomer length and number of connections in the cluster using matplotlib (Hunter and engineering 2007) library. The histograms are generated and represented as html document. pyTanFinder is licensed under the MIT License and is available from GitHub repository (https://github.com/Kirovez/pyTanFinder).

### Slide preparation

For chromosome and nucleus preparation, the Gransden strain of *P.patens* was grown in Knop medium with 500 mg/L ammonium tartrate with 1.5% agar (Helicon, Moscow, Russian Federation) in a Sanyo Plant Growth Incubator MLR-352H (Panasonic, Osaka, Japan) with a 16-hour photoperiod at 24 °C and 61 μmol/m^2^s. Gametophores at different stages (green – light green sporophyte colors) were used for analyses. Chromosome preparation was performed according to the “SteamDrop” protocol (Kirov et al. 2014) with modifications described earlier (Kirov et al. 2015). Briefly, young sporophytes were collected and fixed in Carnoy’s solution (3:1, ethanol/acetic acid) for 3 h at room temperature and stored at −200 °C in 70% ethanol. The fixed material was washed twice in distilled water for 30 min and in 100 mM Citric buffer (pH 4.8). Then, the sporophytes were transferred into the enzyme mixture and incubated for 3 h at 37 °C. The 0.6% enzyme mixture containing Pectolyase Y-23 (Kikkoman, Tokyo, Japan), Cellulase Onozuka R-10 (Yakult Co. Ltd., Tokyo, Japan) and Cytohelicase (Sigma-Aldish Co.LLC, France), was prepared in 0.1 M citric buffer (pH4.8). Slides were prepared using a 1:1 (ethanol/acetic acid) mixture as the first drop and 100% acetic acid as the second drop. Then, slides were additionally incubated for 15–30 s in a drop of 60% acetic acid at 42 °C. One slide per cell suspension was checked by DAPI (100 µg/ml, 4' 6-diamidino-2-phenylindole) staining and mounted in Vectashield (Vector Laboratories, Burlingame, CA).

### NGS sequencing of the moss genome

Isolated DNA was used in NGS sequencing. A sequencing library was prepared by the NEBNext ultra DNA Library Prep Kit for Illumina (New England Biolabs, UK). After preparation of the samples, the libraries were analyzed using Qubit (Invitrogen) and 2100 Bioanalyzer (Agilent Technologies). Amplification of the samples was performed according to the protocol (Illumina) using MiSeq. Raw Illumina fastq files were de-multiplexed, quality filtered and analyzed using FastQC (Schmieder and Edwards 2011). RepeatExplorer tool (Novak et al. 2013) was run with default settings taking 500000 randomly selected single end reads (>100 bp) as input.

### Fluorescence in situ hybridization (FISH) and microscopy

FISH was performed as previously described (Kirov et al. 2017) using TAMRA-labeled oligo probes synthesized by Evrogen (Table 1).

**Table 1.** Oligo probes on TRs used in FISH experiments.

ID	Sequence
17_50	(TAMRA)-AACCTTCTAGAAGAGAAGTTT
21_215	(TAMRA)-ACTTCCAGAGAGCATCGGCAA
602_86	(TAMRA)-AAGTGATGAACAAAATTTCTC
04_78	(TAMRA)-AACTTGCATTCTTCATTTTCA
592_108	(TAMRA)-ATTTCTTAGAAAATACGTTCT
20_76	(TAMRA)-AGTCCCGTCGCGAGTCCCGGA
19_95	(TAMRA)-ATAATTCTATCGGTTATGTTT
05_92	(TAMRA)-AATAATAGTAAAAGTTATAGC
21_43	(TAMRA)-ACCTTCAAGTGGACCTTAGTA
01_31	(TAMRA)-AATCAGCTCGAGTCGAGCTGA
08_44	(TAMRA)-AGCTGATGGCAGGTAAGGGAG
02_27	(TAMRA)-CTTCCGTCTTGGATCCGGAAT
08_217	(TAMRA)-AAAGTAGATCTAAAAATAAAA
05_178	(TAMRA)-ACACGAAACTCACAACTTACT
21_43	(TAMRA)-ACCTTAGTGGAGAAGTTCTGC
18_62	(TAMRA)-AGGGGAGTTTTCAAGTTTTTG
10_116	(TAMRA)-ATTGGAGAAGTATCATTGTAA
16_64	(TAMRA)-ATCGAAGAGCTAGCTTCAAGC
1004_43	(TAMRA)-AGAGAAGTTCTGTCCTTGCCT

### qRT-PCR

Total RNA from protonemata tissue was isolated according to Cove et al. 2000. The RNA quality and quantity were evaluated by electrophoresis in an agarose gel with ethidium bromide staining. The exact concentration was measured using the Quant-iT RNA Assay Kit, 5–100 ng on a Qubit 3.0 (Invitrogen, US). The cDNA for RT-PCR was synthesized using the MMLV RT Kit (Evrogen, Russia). Primers (Table 2) were designed by the Primer 3.0. qRT-PCR with actin gene primer pairs was used as a positive control, whereas qRT-PCR with MQ and DNAse-treated RNA was used as a negative control. RT-PCR was performed using the qPCRmix-HS SYBR system and SYBR Green I (Evrogen) dye on a LightCycler® 96 (Roche, Mannheim, Germany). qPCR was performed in three biological and three technical replicates.

**Table 2.** Primers used for qRT-PCR amplification of PpNATR76 transcripts.

Gene id	Forward	Reverse
Pp3c20_303V3.1	ATGGAGCGGGACAAGAGG	GAGTCCCGACCTCTGGCG
Pp3c20_283V3.1	CCCCCGCCAAAAATGGTTAC	CGGGACAAGGAAGAGGAGGA
Pp3c19_9271V3.1	ACTGGGCTCAAAGAAGGCAG	AGGAGGAAGAGGAGGAAGGC
Pp3c14_12290V3.2	CCCTAGCCTTTGGTTGCGTT	ACTCTCCCTTGCAATGGTCG
Pp3c4_8299V3.1	GTGTCGGGGTTAGGAAGTGG	TAGCTCTTGGAACTCGCTGC

## Results

### Search for tandem repeats in *P.patens* genome by read clustering and pyTanFinder

To find the TRs in the *P.patens* genome, we used the Tandem Repeat Finder tool (TRF, (Benson 1999)). However, TRF provides all the TRs found in the genome; information about the copy number of individual TR monomers is unavailable. Moreover, the TRF output is redundant and it is difficult to manually handle it to find high-copy TRs that possess a certain monomer length and copy number. To overcome these obstacles we designed a python pipeline that we called pyTanFinder (https://github.com/Kirovez/pyTanFinder). It is a user-friendly command line tool to run TRF and parse the results followed by clustering of similar tandem repeats. The output of this program is a FASTA file of all tandem repeats and a table containing unique TR sequences with the estimated abundance in the genome. In addition, pyTanFinder also generates a html report containing histograms of the distribution of the TR monomer size and number of connections of each monomer into an individual cluster. We applied the pyTanFinder pipeline to the *P.patens* (v3.3) genome sequence. We identified 1518 TRs with a minimum length of genome occupy 1000 bp. Because TRs can be collapsed during genome sequence assembly, we performed low-coverage Illumina DNA sequencing followed by *de novo* annotation of TRs in next generation sequencing data using the RepeatExplorer tool (Novak et al. 2013). The clustering of the genomic reads did not reveal any clusters with a ring or globular shape that both corresponded to high-copy TRs. We then compared DNA sequences produced by the pyTanFinder pipeline and RepeatExplorer to find TRs with high copy number in both datasets. 19 TRs that were found in both datasets were used for further analysis (Table 3).

**Figure 1.**
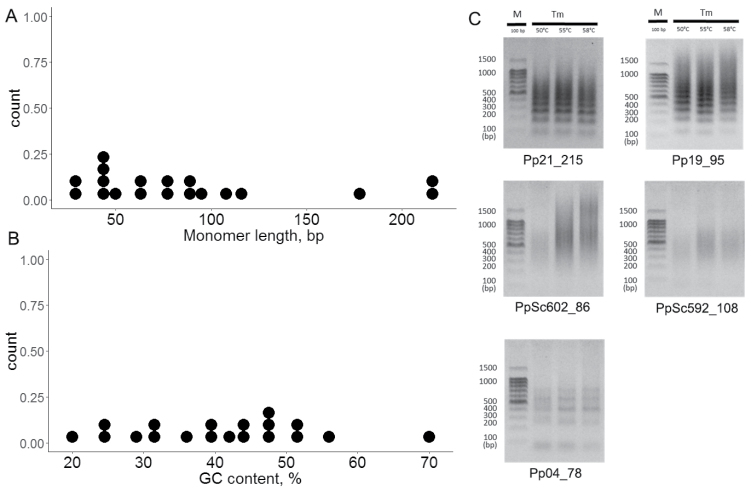
Features of 19 TRs. **A** Monomer length distribution **B** GC content distribution **C** Electrophoresis of PCR products from 5 TRs.

The monomer length of the TRs ranged from 27 to 217 bp (Fig. 1A) and the GC content varied from 20 to 70% (Fig. 1B).

**Table 3.** General information about identified tandem repeats used for FISH analysis.

Id	Monomer length, bp	Repeat Explorer cluster	Abundancy, bp	Sequence
Pp17_50	50	10	285023	GAACCTTCTAGAAGAGAAGTTTCTAGAACCTTCTAGAAAAGAAGCCTCTG
Pp21_215	215	309	156974	CACTTCCAGAGAGCATCGGCAATTTGAACTCTCTTGTGGAGTTGAATTTGTATAGATGTCGATCCTTGAAGGCACTTCCAGAGAGCATCGGCAATTTGAACTCTCTTGTGGAGTTGAATTTGTATGGATGTCGATCCTTGAAGGCACTTCCAGAGAGCATCGGCAATTTGAACTCTCTTGTGAAGTTGAATTTGGTAGATGTCGATCCTTGAAGG
Pp602_86	86	2626	60915	AAGTGATGAACAAAATTTCTCATTTTGCCAAGTGATGAACAAAATTTCTCATTTGCCAAGTGATGAACAAAATTTCTCATTTTGCC
Pp04_78	78	340	38748	CAACTTGCATTCTTCATTTTCATGCTCAACTTACATTCTCTATTTCCATGCTCAACTTGCATTCTCTATTTCCATGCT
Pp592_108	108	1758	34258	ATTTCTTAGAAAATACGTTCTAAATGCAAAGATACAATTTCTTAGAAAATACGTTCTAAATGCAAAGATACAATTTCTTAGAAAATACGTTCTAAATGCAAAGATACA
Pp20_76	76	226	22386	TCCCAGTCCCGTCGCGAGTCCCGGACTTCCTCCTCCTCTTCCTTGTCCCGCCGCGACTCCCTAGTCCCGGCGCGAG
Pp19_95	95	363	18717	ATAATTCTATCGGTTATGTTTAAGGTATTCAAGATATTATCATATACCAATGAATGAATAATGTGCCATTGCCCACCCAAATATTGGAGTTTACC
Pp05_92	92	209	13907	CCTCTAATAATAGTAAAAGTTATAGCAATAAATAATAATTATCAGACTTCCAATAATAGTAAAATTTATAGCAATAAATAATAATTATCGGA
Pp21_43	43	1161	10324	CCTTGCCTTCACCTTCAAGTGGACCTTAGTAGAGAAGTTTTGT
Pp01_31	31	178	5381	AATCAGCTCGAGTCGAGCTGATTTGCTTCTC
Pp08_44	44	193	3978	AGCTGATGGCAGGTAAGGGAGATTGCATGAATCAGCTCGAGTCG
Pp02_27	27	118	3648	CTTCCGTCTTGGATCCGGAATTGGCTC
Pp08_217	217	227	3472	TTTCTTAAAGTAGATCTAAAAATAAAAGTTTTGTCAAAAAAGTAGGCTTTGCTAAGTGATGACTAGAAGTGATTTCTATGTTTGAAGATGCAAAGCTCCTCTTGTTTGTTGTTAAGAAGTATAATTTACTAAAATAAGTTATTAAATAAACAGGAAAATCAAGACGTAAGATTCCTCACAAGATTTGGGATTTACTTCAGAAAACCAACAATTCAAG
Pp05_178	718	2110	2848	CACACGAAACTCACAACTTACTCCGCACACAACTGATCGTCGACAACGTCGTAAAGCAAGGCAACATCAGTGACAACAACGGGGAATCCTACAGTTTTGTGTCCACAACCTTCTCCTCACAAGTGAGATGAGGAACCCATCCGATATCTTTGTGAGGGAGTGATGATACCGGAGGAAT
Pp21_43	43	1161	2648	GTGGACCTTAGTGGAGAAGTTCTGCCCTTGCCTTCACCTTCAA
Pp18_62	62	13	2608	AGGGGAGTTTTCAAGTTTTTGCAAGGTTACTAGTTCGGTTTCATTGGAGGTTTTTGAAGATC
Pp10_116	116	115	1619	ATTGGAGAAGTATCATTGTAAAGCAAGACTATGGAGGTATAAAAAGGGAGGTACATTTACAAGATATAGATGCCTTTGATTTAAGTTTTATTAAAAAAAAAAAAAAAAAAAAAAAA
Pp16_64	64	116	1572	GGGGTTTTTTGGATCGAAGAGCTAGCTTCAAGCTCTTTTCAAGGTCACTAGGTTGGTTTCATTA

According to the pyTanFinder results, 7 (37%) TRs have high (>18000 bp, hcTRs) and 12 (63%) TRs have low (<15000 bp, lcTRs) total abundance. We were able to design primers for 5 hcTRs and obtained ladder-like or smear PCR products (Fig. 1C) that are known characteristic features of TRs (Kirov et al. 2017). Only 8 of 19 identified TRs (trTRs) were similar to the RepeatExplorer contigs from the top 200 clusters, whereas the other TRs were similar to low abundant repeat clusters. Interestingly, the pyTanFinder total abundance data did not correlate with the RepeatExplorer genome proportion data, as only 2 of the trTRs were in set of hcTRs (Table 1). Therefore, based on two approaches (pyTanFinder and RepeatExplorer) we were able to identify two sets of TRs in the moss genome that have a high and low copy number.

### FISH localization of tandem repeats in *P.patens*

**Figure 2.**
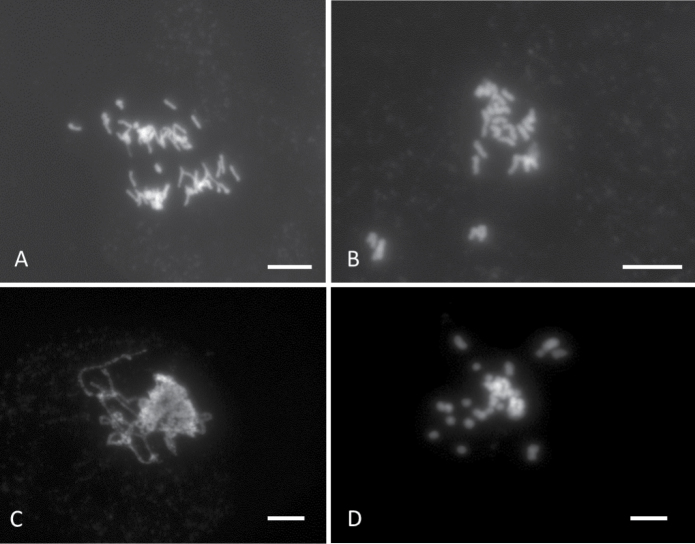
Mitotic and meiotic chromosomes of *P.patens* after DAPI staining. Anaphase (**A**), 1n ((**B**) protonema, n=27) and 2n ((**D**) sporophyte, 2n=54) metaphases and pachytene (**C**) stages. Scale bar: 5 µm.

We used FISH to determine whether the identified TRs occupy large clusters in the moss genome. A molecular cytogenetic approach to visualize DNA sequence loci on chromosomes and nuclei is challenging for bryophites (Rosato et al. 2016). To perform a pilot FISH experiment, we optimized the “SteamDrop” protocol (Kirov et al. 2014) for the preparation of the moss chromosome. Different types of material were used including protoplast, protonemata and unmatured sporophyte. No metaphase chromosomes were observed when protoplasts were used. The chromosome preparation from protonemata and unmatured sporophyte tissues resulted in a very low number of cells in the metaphase stage. Even the pretreatment of protonema tissue with different cytostatic chemicals (colchicine (3–4 h incubation in 0.05 – 0.3%), 1-bromnaphtalene (overnight incubation in saturated solution), and amiprofos-methyl (3–4 h incubation in 5 μM solution)) did not improve the results. The examples of anaphase, 1n (protonema, n=27) and 2n (sporophyte, 2n=54) metaphases as well as pachytene chromosomes after 4',6-diamidino-2-phenylindole (DAPI) staining are shown in Fig. 2.

**Figure 3.**
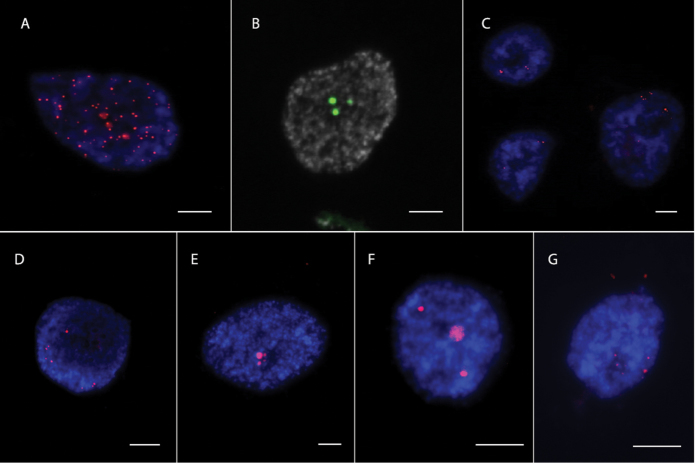
Results of FISH with labeled probes designed on Arabidopsis-type telomere repeat (**A**), 45S rDNA (**B**) and 5 identified TRs: Pp602_86 (**C**), Pp21_215 (**D**), Pp20_76 (**E**), Pp19_95 (**F**) and Pp592_108 (**G**).

We designed 19 TAMRA oligonucleotide probes to perform a nuclei-FISH assay. To validate that the obtained slides were suitable for FISH experiments, we used known tandemly organized sequences, Arabidopsis-type telomeric repeat ((TTTAGGG)n) and 45S rDNA, as positive controls. FISH experiments revealed many dot-like (Fig. 3A) and few distinct (Fig. 3B) signals for telomere and 45S rDNA probes, respectively, which suggested that the slides were suitable for FISH analysis in moss. We then performed nuclei-FISH experiments for 19 moss TRs. These experiments revealed 5 TRs for which FISH signals were detectable on the nuclei (Fig. 3).

**Figure 4.**
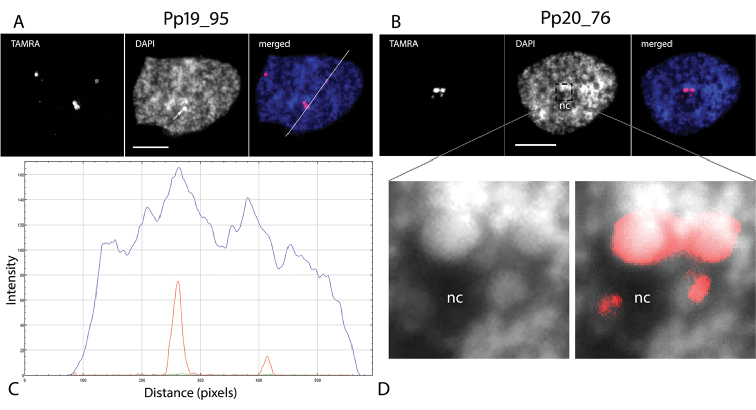
Nuclear organization of Pp19_95 (**A, C**) and Pp20_76 (**B, D**) TRs. **A** and **B** picture series shows fluorescence on DAPI and TAMRA channels and merged pictures **C** RGB profile of the nucleus; blue and red lines show the pixel intensity for two Pp19_95 FISH signals and DAPI staining, respectively **D** Digitally zoomed in part of the nucleus with red Pp20_76 FISH signals. nc marks the nucleolus. Scale bar: 5 µm.

Three repeats (Pp602_86, Pp21_215, Pp592_108) gave several signals that occupied two distinct territories in the nucleus. FISH signals from one TRs, Pp19_95 (95bp monomer size), were associated with heterochromatin regions of the nucleus (Fig. 4A, C) detected by DAPI. FISH signals from another TR, Pp20_76, were located at one nuclear region that was in close proximity to the nucleolus (perinucleolar region), which can be well-distinguished by DAPI staining (Fig. 4B). In contrast to Pp19_95 TR, the DAPI profile from Pp20_76 hybridization loci does not show any clear differences from neighboring nuclear regions. A closer look at the FISH signals shows that Pp20_76 loci are organized as a droplet-like structure (Fig. 4D).

Thus, nuclei FISH analysis of 19 TRs identified by pyTanFinder pipeline showed 5 TRs with pronounced signals. Moreover, one (Pp19_95) of the repeats was associated with heterochromatin structures while another one (Pp20_76) was associated with perinucleolar bodies. The 5 TRs were used for further analysis.

### Location of the TRs in moss genome

**Figure 5.**
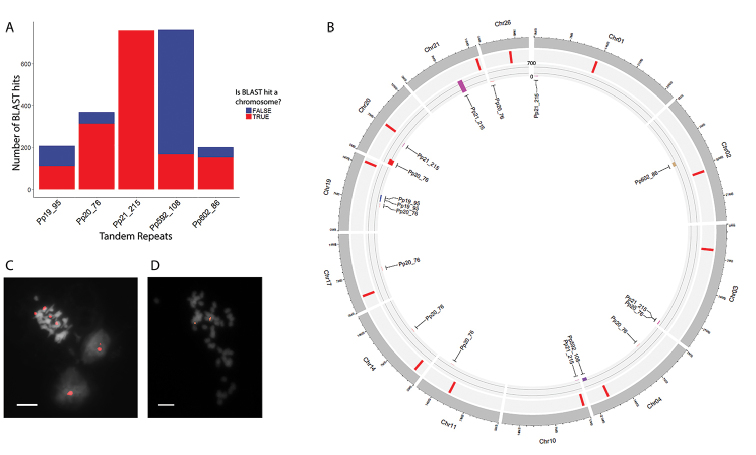
Chromosome location of 5 TRs. **A** Bar plot showing the number of BLAST hits derived from scaffolds and chromosome sequences **B** Circos plot: the inner layer corresponds to the bar plot showing the number of BLAST hits of the TRs on the chromosomes; FISH localization of Pp20_76 (**C**) and Pp602_86 (**D**). Scale bar: 5 µm.

To integrate our data with the *P.patens* genome sequence, we mapped 5 TRs back to the assembled *P.patens* genome sequence and estimated the genomic distribution of the TRs. Up to 45% (for Pp19_95) of BLAST hits belonged to the sequences that were not included in any assembled chromosomes (scaffolds), suggesting a challenge in the assembly of the genomic regions carrying the TRs (Fig. 5A). All BLAST hits were distributed along 12 *P.patens* chromosomes. The Pp602_86, Pp21_215, Pp20_76, Pp19_95 and Pp592_108 TRs had 1, 5, 8, 2 and 1 loci in the assembled genome, respectively. Most of the identified loci contained only a few monomers; each of the repeats possessed a single locus with a high (up to 700) number of tandemly organized repeats including Pp21_215 (Chr21), Pp602_86 (Chr02), Pp592_108 (Chr01), Pp19_95 (Chr19) and Pp20_76 (Chr20). Two TRs, Pp21_215 and Pp20_76, had a bias toward distal parts of the chromosomes, with 60% (3) and 34% (3) loci located at the ends of the assembled chromosomes, respectively (Fig. 5B). A comparison of the putative centromere (RLC5 retrotransposon, Lang et al. 2018) and the TR locations revealed co-localization of 2 Pp21_215 (25%) loci on Chr10 and Chr20 with the RLC5-enriched regions, suggesting possible pericentromeric localization of this TR.

To further verify the results of nuclei-FISH and bioinformatics mapping, we performed FISH on moss chromosomes using two probes, Pp602_86 (single locus) and Pp20_76 (multiple loci). Although the chromosome preparation protocol needs to be further improved for *P.patens*, we were able to identify FISH signals from Pp20_76, located at the ends of two chromosome pairs, and from Pp602_86, located in the proximal positions of one chromosome pair (Fig. 5). FISH results for Pp60_86 correlated well with bioinformatics analysis which also showed a single locus on chromosome 2. In contrast, Pp20_76 has multiple loci in the moss genome assembly; two loci were revealed by FISH. One of the explanations of this discrepancy in bioinformatics and *in situ* experiments may be the limitation of FISH method sensitivity. The sensitivity of FISH does not allow to physically map the DNA sequences if they occupy on the chromosomes less than 3–10 Kb (Valárik et al. 2004, Khrustaleva and Kik 2001). Therefore only the longest Pp20_76 array, located on Chr20, could potentially be visualized by this method. In addition, the FISH signals we observed were located at the end of the chromosomes, which is also in concordance with bioinformatics search. At the same time, a second FISH signal may be derived from Pp20_76 locus that was probably not well assembled. Therefore, the genomic mapping results together with FISH results provided evidence that the TRs that were detected occupied long clusters in the moss genome and allowed further integration of the TR location with the genomic context data available for *P.patens* (Lang et al. 2018).

### Pp20_76 is located in actively transcribed chromatin

**Figure 6.**
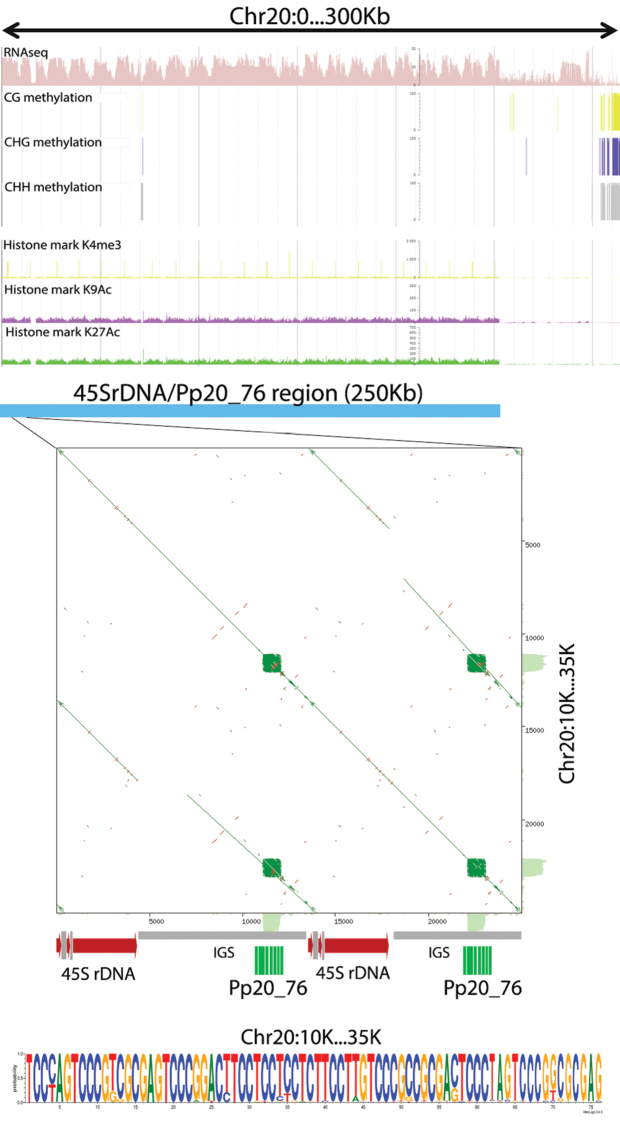
Genomic organization and epigenetic landscape of 45SrDNA/PpNATR76 locus. Top panel is a snapshot of CoGe GBrowser for *P.patens* (https://genomevolution.org/) . Logo picture from multiple alignment of 200 PpNATR76 monomers is shown at the bottom.

Because of the special location of Pp20_76 in the nucleus (near nucleolus) and the detected nucleus bodies enriched by this TR, we named this TR as PpNATR76(76 bp ***P. p****atens* peri**N**ucleolar **A**ssociated **T**andem **R**epeat) and analyzed it further. The alignment of 200 PpNATR76 sequences found in the moss genome showed a high conservation level between monomers. In addition, sequence analysis of the consensus PpNATR76 monomer revealed a long polypyrimidine track ((CCT)_n_ motif). To determine why PpNATR76 DNA was located proximal to the nucleolus, we mapped the 45S rDNA to the moss genome. Using *A.thaliana* 45S rDNA gene (GenBank: X52320.1), we found two minor rDNA loci in the moss genome located on chromosomes 18 and 26 and one major rDNA locus on chromosome 20. The chromosomal location of 45S rDNA and PpNATR76 were identical on chromosomes 20 and 26, where they occupied c. 250Kb and 16Kb regions, respectively. Moreover, a detailed analysis of the loci revealed that PpNATR76 was located between 45S rDNA genes, in the IGS regions (Fig. 6A). Using the data available for moss, as a model organism, we checked the DNA and histone epigenetic landscape in the largest cluster on Chr20. We found a clear reduction in CG, CHG and CHH DNA methylation in the 45S rDNA/ PpNATR76 region (Fig. 6). In addition, the level of ‘active’ (H3K4me3, H3K9Ac, H3K27Ac) histone marks was significantly higher in this region compared with the flanking ones (Fig. 6). We also checked RNAseq data and found high level of RNAseq read coverage for this region, as expected for rDNA loci (Fig. 6).

### PpNATR76 is transcribed into lncRNAs

Because of the transcription activity of the PpNATR7-occupying region, our next aim was to find *P.patens* transcripts possessing the PpNATR76 TR. This analysis revealed 16 transcripts whose genes were located on 5 chromosomes (Chr20, Chr19, Chr4, Chr17, Chr14). Only 4 of the transcripts possessed annotated canonical ORFs (Pp3c19_9270V3.1, p3c19_9271V3.1, Pp3c4_8299V3.1 and Pp3c14_12290V3.1). Pp3c14_12290V3.1 was the only transcript that had ORF with homology to known proteins and was annotated as NADH:ubiquinone reductase, whereas predicted proteins from other PpNATR76 possessing transcripts did not show any homology to known proteins. These data suggested that the PpNATR76 transcripts mostly belonged to lncRNAs. To assess the robustness of the results, we performed a quantitative RT-PCR (qRT-PCR) validation of 5 PpNATR76 transcript genes (Pp3c20_303V3.1, Pp3c19_9271V3.1, Pp3c20_283V3.1, Pp3c14_12290V3.2, Pp3c4_8299V3.1) using protonemata RNA samples. For this experiment, DNA was taken as a positive control, whereas extracted RNA and MQ were negative controls. We then calculated the difference between the Cq values of pure RNA (DNA contamination control) and cDNA specific amplification. The results of qRT-PCR showed that all transcripts were expressed on detectable levels of > 5 delta. In addition, for 3 out of 5 genes, sense as well as antisense transcriptions were observed, whereas for two genes (Pp3c20_283, Pp3c14_12290) only one-way directed transcription was detected. Collectively this data proved the existence of the pPNATR76 transcripts in somatic cells and strongly suggested that PpNATR76 was transcribed as part of both protein coding and lncRNAs.

## Discussion

TRs with different monomer sizes are integral parts of most eukaryotic organisms, in which they are involved in diverse biological processes. Although many efforts have been made to understand the genomic organization, structure and evolution of TRs, their functions in a cell are still poorly understood. Here, we performed a pioneering identification and FISH verification of satellite repeats, forming a long array in the genome of the model plant, *P.patens*. We developed a pipeline, pyTanFinder, and identified 19 TRs, of which 5 TRs produced FISH signals. We found both heterochromatin associated and transcribed TRs. Genomic and transcriptomic analyses identified IGS-associated moss TR, PpNATR76, which was sequestered in the perinucleolar space and transcribed as a part of lncRNAs.

### pyTanFinder pipeline identified heterochromatin located satellite DNA sequences in moss

Advances in genome sequencing and bioinformatics approaches in the last decades has triggered the progress in satellite repeat isolation (reviewed by (Lower et al. 2018)). We explored the satellitome of the model plant, *P.patens*, using our pyTanFinder pipeline and repeat library generated by RepeatExplorer (Novák et al. 2013). Although a large number of TR identification tools have been developed (reviewed by (Lower et al. 2018), the pyTanFinder pipeline can be very useful if the available full genome sequence is highly fragmented. It is very common for satellite repeats to collapse during genome assembly (Saint-Oyant et al. 2018). Therefore, the identification of a TR in a single locus produced by some tools may lead to some spurious results. This limitation is overcome in the pyTanFinder pipeline by clustering of similar TRs identified across all chromosome and scaffold sequences followed by calculation of the TR abundance based on all sequences in a cluster. This approach also makes it possible for pyTanFinder to be applied for the identification of satellite repeats in long-read single molecule real time genome sequencing data generated by modern PacBio and Oxford NanoPore platforms. Our preliminary results obtained on PacBio data of *Aegilopstaushii* Coss., 1850 (SRA archive at NCBI: SRX3098055) supports this suggestion (data not shown). The pioneering satellite DNA identification and its FISH mapping in the moss nucleus performed in this study resulted in a set of cytogenetic markers that can be useful for future genomic and cytogenetic data integration. As shown in many other plants, the integration of chromosomal and sequence data may help to shed more light on genome evolution and to correct genome assembly ((Fransz et al. 2016, Kirov et al. 2015, Saint-Oyant et al. 2018, Shearer et al. 2014)). Molecular cytogenetic techniques, such as FISH, have never been applied to mosses; therefore, the chromosome preparation and FISH mapping procedures described in this study are important for further improvement of the *P.patens* genome assembly and annotation. Interestingly, recent (Lang et al. 2018) as well as earlier works (Melters et al. 2013) have shown low TR abundancy in the genomes of basal plants. In concordance with this observation, Lang et al. (2018) also observed a lack of clear heterochromatin regions on nuclei that typically contain TRs. Although we also did not observe large heterochromatin blocks, our slide preparation procedure allowed us to identify some small heterochromatin blocks in the moss nucleus (Figs 3, 4). In addition, the pyTanFinder pipeline allowed us to isolate at least one TR Pp19_95, which was enriched in the identified heterochromatin regions. Moreover, this repeat exhibits strong DNA methylation compared with that of the neighboring regions, which also suggested that it was located in the heterochromatin. It would be interesting to check in the future whether the heterochromatin organization is similar between basal plants and angiosperms.

### Intergenic 45S rDNA spacer is a source of satellite non-coding transcripts: a principle that is conserved from first land plants to human

We found one IGS-related satellite repeat, named PpNATR76, that had several distinguishable features at the genome and transcriptome levels: 1) its DNA occupied distinct perinucleolar-associated chromatin bodies and most of its copies were located in IGS 45S rDNA spacer; 2) its DNA was hypomethylated and associated histones were enriched in ‘active’ chromatin marks and 3) it was transcribed into lncRNAs. The number (four signals for diploid nucleus used in this study) of PpNATR76 FISH signals was in agreement with previously observed 1–2 rDNA loci in moss and other bryophytes (Berrie 1958a, b, Rosato et al. 2016, Sone et al. 1999). As this TR was a part of the IGS region and its FISH signals on the nucleus (Fig. 4B, D) were identical to 45S rDNA (Fig. 3B), we supposed that the observed PpNATR76 perinucleolar bodies were knob-like rDNA chromatin. From a first glance, this was not congruent with ‘active’ histone marks and the almost absence of DNA methylation in the 45S rDNA/IGS/PpNATR76 region because the knob structure consisted of heterochromatin. However, condensed knobs and decondensed transcriptionally active rRNA genes are interspersed in one NOR region (Pontes et al. 2003). Indeed, we also found high concentration of ‘inactive’ chromatin marks in this region of the *P.patens* genome (H3K9me2, H3K27me3, data not shown). Because of the identity of ‘active’ and ‘inactive’ 45S rDNA sequences, the bioinformatics mapping of Chip-seq reads to the genome is not able to distinguish them and leads to erroneous results when ‘active’ and ‘inactive’ chromatin marks co-occurred. Therefore, PpNATR76 TR is a part of both knob-like (‘inactive’, visualized by FISH) and transcriptionally active (invisible by FISH because of the low local nuclear density of labeled loci and limited FISH sensitivity) chromatin.

Satellite DNA repeats frequently originate in plant IGS DNA and have similar organization between closely related species (Almeida et al. 2012, Falquet et al. 1997, Jo et al. 2009, Lim et al. 2004). However, the PpNATR76 length (76bp) was much shorter than the previously described IGS-associated TRs (>170 bp). IGS-associated short TRs (STR) with a monomer length range from 2 to 12 have also been described in humans (Goodwin and Swanson 2014, Yap et al. 2018). Interestingly, we showed the existence of PpNATR76 containing lncRNAs in moss cell. Recently, Yap et al. (2018) also found multiple STR-enriched lncRNAs (PNCTR) in human cell. In addition, PpNATR76 lncRNAs possess poly-pyrimidine (purine) track, which was also identified in PNCTR RNAs, where it is recognized by pyrimidine tract-binding protein (PTBP1)-specific motifs, allowing it to sequester a significant fraction of PTBP1 in the perinucleolar compartment. Poly-purine stretches were also found in another rDNA IGS-related lncRNA, PAPAS (Bierhoff H et a., 2017, Zhao et al. 2018), in which this motif is involved in forming a DNA-RNA triplex that tethers this lncRNAs to the enhancer region of rRNA genes. The described features make genomic and transcriptomic organization of moss PpNATR76 lncRNAs and human IGS related lncRNAs quite similar. Although future studies of PpNATR76 lncRNAs are required, it can be speculated that the transcription of functionally important satellite-possessing lncRNAs from the IGS region is a conserved principle between plants and humans. Because of the activity of rDNA loci, IGS-related TRs have exceptional location in the genome that promotes their transcription, resulting in the origin of novel classes of lncRNAs. This remarkable feature distinguishes this type of TR from heterochromatin-associated TRs. Our results pose a number of questions about the possible function of PpNATR76 lncRNAs as well as the existence of similar IGS-related lncRNAs in other basal species and angiosperms.

## Conclusions

In this study we extended the list of model plant species for TR studies with a well-known model “basal” plant, *P.patens*, and provided a set of new FISH-verified TRs for further functional and evolutionary analysis in moss. We described a new pipeline pyTanFinder for the identification of TR in fragmented genome sequences and demonstrated the conservation principle of IGS-related TR lncRNA expression between human and early diverged land plants. The results of our work will accelerate further studies of TR biology and function in a plant cell using the model “basal” plant *P.patens*.
